# Observation on Discordant Treatment Response on [ ^18^ F]FDG and [ ^68^ Ga]Ga-FAPI-04 PET/CT in Radioiodine-Refractory Thyroid Carcinoma Treated with Lenvatinib

**DOI:** 10.1055/s-0046-1822663

**Published:** 2026-05-05

**Authors:** Rangat Bagasariya, Sunita Sonavane, Sandip Basu

**Affiliations:** 1 Radiation Medicine Centre, Bhabha Atomic Research Centre, Tata Memorial Centre Annexe, Mumbai, Maharashtra, India; 2 Homi Bhabha National Institute, Mumbai, Maharashtra, India

**Keywords:** differentiated thyroid carcinoma, TENIS syndrome, [ ^18^ F]FDG PET/CT, [ ^68^ Ga]Ga-FAPI-04, lenvatinib, tyrosine kinase inhibitor

## Abstract

**Background:**

Dual-tracer positron emission tomography/computed tomography (PET/CT) using [ ^18^ F]FDG and fibroblast activation protein inhibitor (FAPI) provides complementary metabolic and stromal information in radioiodine-refractory differentiated thyroid carcinoma (DTC). However, response patterns during systemic therapy remain incompletely understood.

**Case:**

A 60-year-old male with papillary thyroid carcinoma (pT2 pN1a pM0) underwent total thyroidectomy and bilateral neck dissection. After defaulting from follow-up, he developed diffuse pulmonary and mediastinal metastases. Following one therapeutic dose of 200 mCi (7.4 GBq) [ ^131^ I], the disease became radioiodine-refractory (thyroglobulin-elevated negative iodine scintigraphy syndrome). Baseline Fluorine-18 Fluorodeoxyglucose Positron Emission Tomography/Computed Tomography ([ ^18^ F]FDG PET/CT) demonstrated hypermetabolic pulmonary and mediastinal metastases. Gallium-68 fibroblast activation protein inhibitor Positron Emission Tomography/Computed Tomography ([ ^68^ Ga]Ga-FAPI-04 PET/CT) performed the following day showed corresponding fibroblast activation protein expression without discordant lesions, although there was a low expression profile of [ ^68^ Ga]Ga-FAPI-04 PET/CT observed at the baseline compared with the [ ^18^ F]FDG PET/CT. After 10 months of lenvatinib (14 mg once daily), [ ^18^ F]FDG PET/CT demonstrated stable metabolic disease according to PERCIST 1.0 criteria, whereas [ ^68^ Ga]Ga-FAPI-04 PET/CT showed marked reduction of FAP expression in mediastinal nodes and several pulmonary nodules. Serum thyroglobulin decreased from 574 ng/mL to 457 ng/mL without anti-thyroglobulin antibody interference. The multidisciplinary team classified the case as stable disease based on persistent FDG avidity.

**Conclusion:**

Discordant response patterns between FDG and FAPI PET/CT highlight the complementary biological information provided by dual-tracer imaging. Reduction in stromal FAP expression did not equate to metabolic remission. Combined interpretation may improve response assessment in radioiodine-refractory DTC.

## Introduction

Thyroid carcinoma is the most common endocrine malignancy, with papillary thyroid carcinoma (PTC) being the most frequent subtype. Regional lymph node metastases are common, and distant metastases occur most frequently in the lungs. 1 Extensive macronodular pulmonary metastases are associated with poorer outcomes. 2

Radioiodine ( ^131^ I) therapy remains the standard treatment for metastatic differentiated thyroid carcinoma (DTC). However, dedifferentiated, radioiodine-non-avid tumor cells may persist and progress, resulting in radioiodine-refractory disease, commonly termed thyroglobulin-elevated negative iodine scintigraphy (TENIS) syndrome. In such cases, systemic therapy with tyrosine kinase inhibitors is recommended. Lenvatinib has demonstrated significant progression-free survival benefit in the phase III SELECT trial. 3 A post-hoc analysis showed improved overall survival in patients with lung metastases, particularly those with lesions measuring 1 to 2 cm. 4

Fluorine-18 Fluorodeoxyglucose Positron Emission Tomography/Computed Tomography ([ ^18^ F]FDG PET/CT) plays an important role in staging and response assessment in TENIS. High FDG uptake reflects tumor dedifferentiation and is associated with adverse prognosis. 5 6 Fibroblast activation protein (FAP), expressed in cancer-associated fibroblasts, represents a novel stromal target. ^68^ Ga-labeled fibroblast activation protein inhibitor (FAPI) tracers enable noninvasive assessment of tumor stroma and have shown promising results in thyroid cancer. 7 FAPI imaging also has potential theranostic implications with ^177^ Lu-labeled compounds. 8

The authors report a case of TENIS syndrome with pulmonary and mediastinal metastases demonstrating discordant response on serial [ ^18^ F]FDG and Gallium-68 FAPI Positron Emission Tomography/Computed Tomography ([ ^68^ Ga]Ga-FAPI-04 PET/CT) following lenvatinib therapy.

## Case Report

A 60-year-old male presented with anterior neck swelling. Fine-needle aspiration cytology revealed malignancy. He underwent total thyroidectomy with bilateral neck dissection. Histopathology showed multifocal PTC with gross extrathyroidal extension and multiple metastatic lymph nodes (pT2 pN1a pM0). Postoperative serum thyroglobulin (Tg) was 663 ng/mL.

The patient defaulted from follow-up for 33 months and later presented with breathlessness and elevated Tg (1,356 ng/mL) without anti-Tg antibody interference, despite adequate thyroid hormone suppression. CT thorax demonstrated multiple bilateral pulmonary nodules. Biopsy confirmed metastatic PTC.

He received 200 mCi (7.4 GBq) of [ ^131^ I] therapy. The post-therapy scan showed uptake in the neck and diffusely in both lungs. Seven months later, a low-dose (3 mCi [111 MBq]) [ ^131^ I] whole-body scan showed no residual iodine-avid disease, while CT revealed persistent pulmonary nodules. Tg remained elevated at 574 ng/mL without antibody interference. The disease was classified as TENIS.

**Fig. 1:**
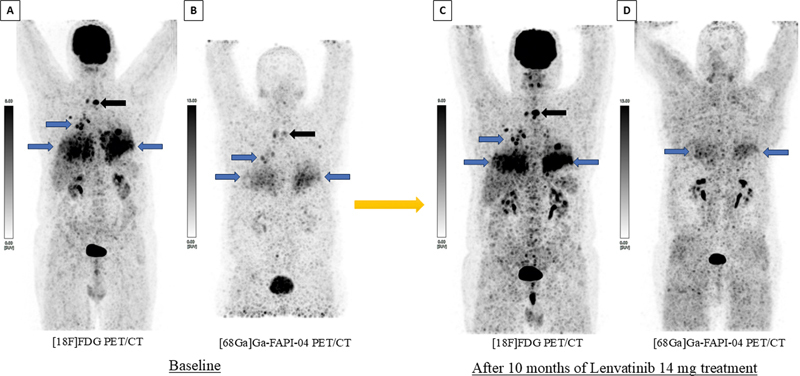
Baseline maximum intensity projection (MIP) images of ( **A** ) [ ^18^ F]FDGPET/CT and ( **B** ) [ ^68^ Ga]Ga-FAPI PET/CT demonstrate diffuse [ ^18^ F]FDG uptake and fibroblast activation protein expression in bilateral pulmonary metastases ( *blue arrows* ) and mediastinal lymph nodes ( *black arrow* ). After 10 months of lenvatinib therapy, follow-up of ( **C** ) [ ^18^ F]FDG PET/CT shows stable metabolic activity, whereas ( **D** ) [ ^68^ Ga]Ga-FAPI PET/CT demonstrates marked reduction in FAP expression in pulmonary and mediastinal lesions. [ ^18^ F]FDG PET/CT, Fluorine-18 Fluorodeoxyglucose Positron Emission Tomography/Computed Tomography; FAP, fibroblast activation protein inhibitor; [ ^68^ Ga]Ga-FAPI PET/CT, Gallium-68 fibroblast activation protein inhibitor Positron Emission Tomography/Computed Tomography.

**Fig. 2:**
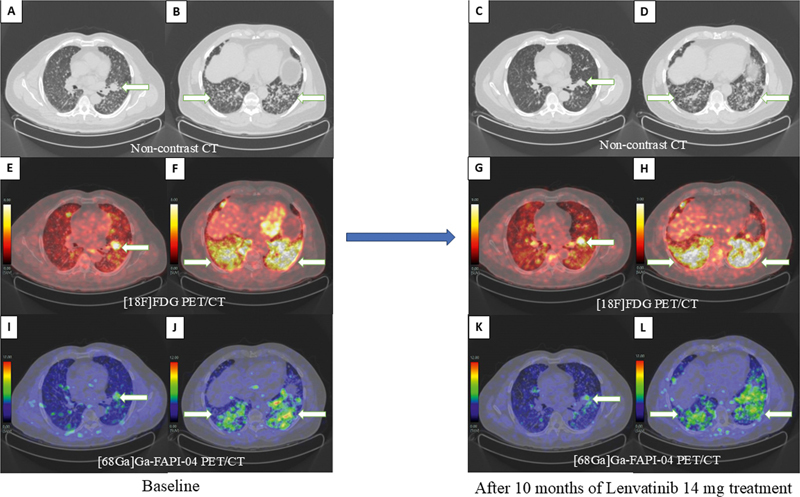
( **A, B** ) Baseline transaxial non-contrast CT and ( **C, D** ) after lenvatinib treatment show structurally stable multiple lung nodules. ( **E, F** ) Baseline [ ^18^ F]FDGPET/CT and ( **G, H** ) after lenvatinib treatment show stable FDG uptake in multiple lung nodules. ( **I, J** ) Baseline [ ^68^ Ga]Ga-FAPI-04 PET/CT and ( **K, L** ) after lenvatinib treatment show resolution of FAP expression in some of the lung nodules. [ ^18^ F]FDG PET/CT, Fluorine-18 Fluorodeoxyglucose Positron Emission Tomography/Computed Tomography; FAP, fibroblast activation protein inhibitor; [ ^68^ Ga]Ga-FAPI PET/CT, Gallium-68 fibroblast activation protein inhibitor Positron Emission Tomography/Computed Tomography.

Baseline [ ^18^ F]FDG PET/CT demonstrated multiple hypermetabolic pulmonary nodules and mediastinal lymph nodes ( Fig. 1A , Fig. 2E and F ). The following day, [ ^68^ Ga]Ga-FAPI-04 PET/CT showed corresponding FAP expression without discordant lesions ( Fig. 1B , Fig. 2I and J ). There was a low expression profile of [ ^68^ Ga]Ga-FAPI-04 PET/CT observed at the baseline compared with the [ ^18^ F]FDG PET/CT.

Lenvatinib was initiated at 14 mg once daily. After 10 months of therapy, follow-up [ ^18^ F]FDG PET/CT demonstrated stable metabolic disease per PERCIST 1.0 criteria ( Fig. 1C , Fig. 2G and H ). In contrast, [ ^68^ Ga]Ga-FAPI-04 PET/CT showed marked reduction of FAP expression in mediastinal nodes and several pulmonary nodules ( Fig. 1D , Fig. 2K and L ). Serum Tg decreased to 457 ng/mL.

Despite stromal response and biochemical improvement, the multidisciplinary tumor board categorized the disease as stable based on persistent FDG avidity. Lenvatinib was continued, and repeat dual-tracer evaluation was planned.

## Discussion

[ ^18^ F]FDG PET/CT is integral to the management of high-risk and radioiodine-refractory DTC and is emphasized in recent American Thyroid Association guidelines. 5 FDG avidity reflects tumor dedifferentiation and adverse prognosis. 5 6

FAPI PET imaging targets cancer-associated fibroblasts within the tumor microenvironment. Lindner et al demonstrated favorable tumor retention and pharmacokinetics of quinoline-based FAPI tracers. 9 However, stromal activity may change independently of tumor glucose metabolism, leading to discordant findings.

Fu et al reported higher maximum standardized uptake value and improved lesion detection with [ ^68^ Ga]Ga-FAPI compared with FDG PET/CT in metastatic DTC, without significant difference in overall diagnostic accuracy. 10 Chen et al reported an 87.5% lesion detection rate in radioiodine-refractory DTC. 11 Ballal et al observed higher detection accuracy of FAPI PET/CT for lung metastases compared with FDG PET/CT, though CT had the highest overall detection rate. 12 In contrast, Hadjitheodorou et al reported superior detection with FDG compared with ^18^ F-FAPI-74 PET/CT. 13 In the present case, there was a low expression profile of [ ^68^ Ga]Ga-FAPI-04 PET/CT observed at the baseline compared with the [ ^18^ F]FDG PET/CT, therefore the possibility of misclassification due to already low baseline FAPI expression was an additional consideration.

Similarly, false-positive FAPI uptake has been described in inflammatory and benign conditions. 14 15 16 Furthermore, although ^177^ Lu-FAPI therapy shows promise, 8 no single FAPI tracer has yet replaced FDG in routine TENIS management.

In this case, FDG PET/CT demonstrated stable disease, while FAPI PET/CT showed reduced stromal activity and Tg levels declined. This suggests that stromal modulation may precede or occur independently of metabolic response. Current literature remains heterogeneous; therefore, FAPI PET/CT alone may not be sufficient for response assessment. A combined dual-tracer approach may provide a more comprehensive evaluation.

## Conclusion

This case demonstrates discordant response patterns on dual-tracer PET/CT in radioiodine-refractory DTC treated with lenvatinib. Reduction in FAP expression did not correspond to metabolic remission on FDG PET/CT. Combined interpretation of metabolic and stromal imaging may improve treatment assessment. Larger prospective studies are needed to define the clinical role of dual-tracer [ ^18^ F]FDG and [ ^68^ Ga]Ga-FAPI PET/CT imaging in TENIS syndrome.
